# A Fast Nonlinear Sparse Model for Blind Image Deblurring

**DOI:** 10.3390/jimaging11100327

**Published:** 2025-09-23

**Authors:** Zirui Zhang, Zheng Guo, Zhenhua Xu, Huasong Chen, Chunyong Wang, Yang Song, Jiancheng Lai, Yunjing Ji, Zhenhua Li

**Affiliations:** 1 School of Physics, Nanjing University of Science and Technology, Nanjing 210094, China; 321113010254@njust.edu.cn (Z.Z.);; 2 Nanjing University of Science and Technology Tangshan Test Center, Nanjing University of Science and Technology, Nanjing 210000, China; 3 Faculty of Mathematics and Physics, Huaiyin Institute of Technology, Huai’an 223003, China; 4 Engineering Research Center of Semiconductor Device Optoelectronic Hybrid Integration in Jiangsu Province, Nanjing 210000, China

**Keywords:** image deblurring, fast nonlinear sparse model, nonlinear sparse regularization, adaptive generalized soft-thresholding

## Abstract

Blind image deblurring, which requires simultaneous estimation of the latent image and blur kernel, constitutes a classic ill-posed problem. To address this, priors based on $L_{2}$, $L_{1}$, and $L_{p}$ regularizations have been widely adopted. Based on this foundation and combining successful experiences of previous work, this paper introduces $L_{N}$ regularization, a novel nonlinear sparse regularization combining the $L_{p}$ and $L_{\infty }$ norms via nonlinear coupling. Statistical probability analysis demonstrates that $L_{N}$ regularization achieves stronger sparsity than traditional regularizations like $L_{2}$, $L_{1}$, and $L_{p}$ regularizations. Furthermore, building upon the $L_{N}$ regularization, we propose a novel nonlinear sparse model for blind image deblurring. To optimize the proposed $L_{N}$ regularization, we introduce an Adaptive Generalized Soft-Thresholding (AGST) algorithm and further develop an efficient optimization strategy by integrating AGST with the Half-Quadratic Splitting (HQS) strategy. Extensive experiments conducted on synthetic datasets and real-world images demonstrate that the proposed nonlinear sparse model achieves superior deblurring performance while maintaining completive computational efficiency.

## 1. Introduction

Recent advances in computer vision have intensified the research focus on image processing, particularly through significant contribution to the development of image deblurring, a fundamental component of low-level image processing. In the context of space-invariant blur kernel modeling, a blurred image y can be mathematically represented as:

(1) B=I⊗k−n

here *B* denotes the blurred image, I denotes the sharp image corresponding to *B*, ⊗ denotes the convolution operator, k denotes the blur kernel, and n denotes the inevitable additional noise. Image deblurring encompasses two distinct categories: non-blind deblurring, where the kernel k is known, and blind deblurring, where the kernel k is unknown. This research addresses the latter.

Blind deblurring algorithms aim to direct the optimization process toward the desired solution, with traditional optimization approaches employing regularization terms to enhance constraints on both the latent image I and blur kernel k, facilitating convergence to the appropriate blur kernel and sharp latent image. The standard formulation of blind deblurring is expressed as:

(2) $$(I,k)=\underset{I,k}{\arg\min}\;F(I,k)+\alpha R_{1}(I)+\beta R_{2}(k),$$

where F(I, k) denotes the data fidelity term; $R_{1}(I)$ and $R_{2}(I)$ denote the regularization terms for the latent image I and blur kernel k, respectively.

Regarding the regularization term $R_{1}(I)$, researchers have developed numerous effective image priors, with gradient sparsity-based priors receiving extensive attention and implementation. For instance, Xu et al. [1] introduced an $L_{0}$ norm-based image smoothing algorithm and subsequently expanded $L_{0}$ regularization to the field of image deblurring [2], revealing that optimizing $L_{0}$ regularization presents a Nondeterministic Polynomial (NP)-hard problem, rendering direct solutions impractical. To address this limitation, Xu et al. [2] developed an unnatural distribution to approximate the $L_{0}$ regularization solution. Concurrently, the compressed sensing community typically addressed this challenge by relaxing the $L_{0}$ norm to one of convex optimization. Earlier approaches commonly replaced the $L_{0}$ norm with that of $L_{1}$ [3,4]; however, the limited sparsity of the $L_{1}$ norm results in performance that fails to match that of $L_{0}$ regularization in image processing applications.

**Figure 1:**
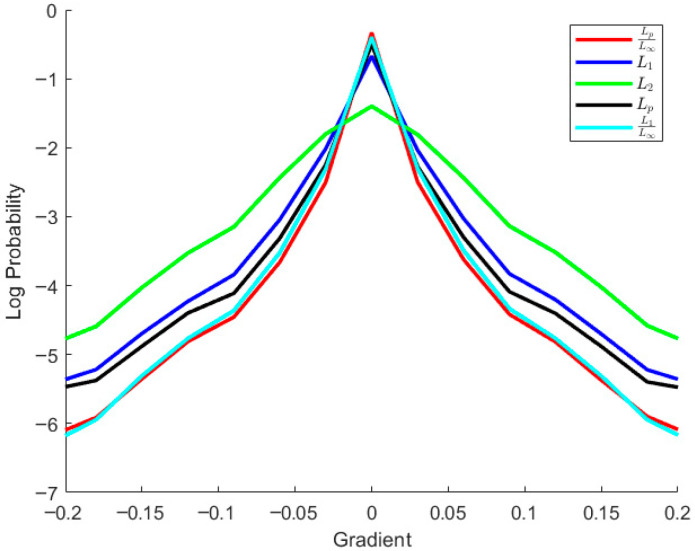
Log probability curves of the gradients of the intermediate latent images obtained from different sparse regularizations on the dataset in [8].

To address this issue, Wang et al. [3,4,5,6] introduced a series of norm-ratio-based regularizations, e.g., $L_{1}/L_{2}$ and $L_{1}/L_{\infty }$, establishing that $L_{1}/L_{\infty }$ could effectively approximate the *L*_0_ norm [7]. Given that the *L*_1_ norm represents a special case of $L_{p}$ norm where *p* = 1, we extend Wang et al.’s work by generalizing $L_{1}/L_{\infty }$ regularization into a broader framework: $L_{p}/L_{\infty }$ (0 < *p* < 1) regularization (termed nonlinear sparse regularization, denoted as $L_{N}$ regularization). To evaluate the effectiveness of $L_{N}$ regularization, we perform a sparsity analysis of various regularizations using gradient distribution statistics, as presented in Figure 1. The results indicate that the proposed $L_{N}$ regularization achieves superior sparsity, offering preliminary evidence of its advantage over $L_{1}/L_{\infty }$ regularization.

Due to its high sparsity, the $L_{0}$ gradient prior exhibits strong filtering capabilities against harmful artifacts in blurred images, although this restricts its performance when processing detail-rich images. To overcome this limitation, researchers have developed an $L_{0}+X$ deblurring prior framework that combines the $L_{0}$ gradient prior with complementary prior terms, a hybrid approach which aims to filter detrimental artifacts effectively while preserving fine image structures. Current *X*-prior terms frequently incorporate various patch-based image priors [9,10,11,12,13]; however, these methods encounter challenges due to their high computational complexity, excessive resource consumption, and low operational efficiency. In response, Chen et al. [14] introduced an enhanced sparse model utilizing the $L_{1}$ gradient prior as the *X*-term, substantially improving computational speed. Building on this work, we incorporate the proposed $L_{N}$ regularized gradient prior as the *X*-term and combine it with the $L_{0}$ gradient prior, developing a novel fast nonlinear sparse model.

The main contributions of this paper are as follows:

1. We propose a novel nonlinear sparse regularization ($L_{N}$) that nonlinearly couples the $L_{p}$ norm with the $L_{\infty }$ norm.
2. An Adaptive Generalized Soft-Thresholding (AGST) algorithm is developed to optimize the $L_{N}$ regularization problem.
3. Building upon $L_{N}$-regularization, we design a novel nonlinear sparse model for blind deblurring and develop an efficient optimization algorithm based on AGST and HQS.

The rest of this paper is organized as follows: Section 2 provides a comprehensive review of existing deblurring methods; Section 3 introduces the proposed nonlinear sparse regularization and the corresponding fast nonlinear sparse model; Section 4 experimentally evaluates the model on synthetic datasets and real-world blurred images; Section 5 analyzes ablation studies in order to validate the components of our fast nonlinear sparse model, including runtime performance tests; and Section 6 gives our conclusion.

## 2. Related Work

Generally, all existing methods can be classified into two categories: optimization-based and deep-learning-based methods. This section provides an overview of these distinct methodologies.

### 2.1. Optimization-Based Methods

Optimization-based image deblurring algorithms originated from the Richardson-Lucy method [15,16]. Subsequently, various approaches emerged, including the Gaussian mixture model (Fergus et al. [17]) and a fast $L_{2}$ norm-based deblurring method (Cho et al. [18]). However, $L_{2}$ regularization demonstrates limited effectiveness in blur kernel estimation due to its insufficient sparsity; therefore, a sparse regularized prior is urgently needed to meet the performance requirements of image deblurring. Yang et al. [19] and Candes et al. [20] proposed several $L_{1}$-norm methods; however, these approaches failed to satisfy the sparsity requirements of the deblurring prior term.

To obtain superior restoration performance, Daniele et al. [21] tried an $L_{p}$ (0 < *p* < 1) gradient prior. Since the kernel estimation based on $L_{p}$ sparse regularization is a non-convex problem, this leads to a hard solution of $L_{p}$ optimization. To address this problem, Daniele et al. [21] transformed the non-convex $L_{p}$ optimization problem into a quadratic optimization problem by taking the log function of the $L_{p}$ regularization term. Gasso et al. [22] and Zou et al. [23] extended Iteratively Reweighted $L_{1}$ minimization (IRL1) [20] to the non-convex problem domain of $L_{p}$ minimization. Rao and Kreutz-Delgado [24] proposed an Iteratively Reweighted Least Squares (IRLS) approach to $L_{p}$ minimization; and She et al. [25] proposed the Iteratively Thresholding Method (ITM), which was only suitable for unconstrained problems. In 2013, Zuo et al. [26] proposed a GST operator to solve the $L_{p}$ minimization problem, and following this, Zuo et al. [27] applied $L_{p}$ regularization to the field of image deblurring.

In pursuit of enhanced norm sparsity during the deblurring process, research focus shifted toward the *L*_0_ norm. Xu et al. [1] introduced an $L_{0}$ image smoothing method in 2011, and building upon this, Xu et al. [2] developed an $L_{0}$ gradient prior by applying $L_{0}$ norm constraints to image gradients, enhancing kernel estimation and large-scale optimization. Extensive research has demonstrated that the generalized $L_{0}$ sparse gradient prior can effectively extract strong edges, and Xu et al.’s [2] groundbreaking work inspired numerous deblurring methods based on $L_{0}$ regularization: Pan et al. [28] implemented $L_{0}$ regularized intensity and gradient prior for text image deblurring, and Li et al. [29] applied $L_{0}$ norm to constrain the blur kernel intensity.

Despite the $L_{0}$-regularization prior’s proven effectiveness in removing harmful artifacts from images and widespread adoption in blind deblurring, it often underperforms when processing images with complex structural details. To address this limitation, researchers developed the + X paradigm, combining the $L_{0}$ gradient prior with supplementary image priors. A representative example is the Dark Channel Prior (DCP) developed by Pan et al. [9], who discovered that sharp images exhibited sparser dark channels compared to blurred ones, combining the DCP with the $L_{0}$ gradient prior and achieving good results. Similarly, Yan et al. [30] extended the applicability of the DCP by combining it with the bright channel prior. Additionally, Eqtedaei et al. [31] developed a deblurring prior based on the difference between local maximum and minimum pixel values within an image region, developing two distinct deblurring algorithms utilizing $L_{1}$ and $L_{0}$ regularization, respectively.

The aforementioned image patch-based priors rely on overlapping patches, which substantially increases their computational complexity. To address this limitation, researchers have explored non-overlapping patches as an alternative approach. Notable examples include the Patch-wise Minimum Pixel (*PMP*) prior proposed by Wen et al. [11] and the Patch-wise Maximum Gradient (*PMG*) prior developed by Xu et al. [13]. These non-overlapping patch priors demonstrate significant computational acceleration while maintaining restoration accuracy; however, despite their improved efficiency, these methods still require individual path processing. Additionally, many patch-based priors necessitate the introduction of large, sparse matrices during optimization, consuming considerable computational resources and reducing algorithmic efficiency.

On the other hand, edge detection-based deblurring algorithms have emerged as a viable technical approach. Joshi et al. [32] implemented direct detection and prediction of latent sharp edges to enhance blur kernel estimation; Cho et al. [18] integrated bilateral filtering, shock filtering, and edge gradient thresholding for salient edges prediction; Xu et al. [33] developed a two-phase robust kernel estimation framework with an effective edge selection strategy; and Pan et al. [34] proposed a self-adaptive edge selection algorithm, while Liu et al. [35] implemented a surface-aware approach. Although explicit edge prediction methods demonstrate validity in blind deblurring, they remain dependent on heuristic filters. These methods therefore tend to amplify noise, potentially compromising the deblurring process and producing over-sharpened images as, furthermore, natural images do not consistently contain salient edges. Additionally, some scholars have begun exploring the integration of learning mechanisms with traditional optimization frameworks [36,37,38].

### 2.2. Learning-Based Methods

In the last decade, the rapid advancement of deep learning technology has prompted researchers to investigate its applications in image deblurring tasks.

In 2015, Sun et al. [39] pioneered the application of Convolutional Neural Networks (CNNs) to non-uniform image deblurring, marking an early successful integration of deep learning techniques. Subsequently, numerous CNN-based methods have emerged. For instance, Chakrabarti et al. [40] modified initial network layer connectivity using multi-frequency decomposition; Gong et al. [41] proposed a CNN-based approach for direct motion flow estimation from blur kernels; Ren et al. [42] designed a Maximum a Posteriori (MAP) deep learning hybrid framework, utilizing dual-branch networks for the alternating optimization of latent sharp images and blur kernels. Feng et al. [43] proposed Ghost-UNet, incorporating lightweight sub-networks for enhanced computational efficiency while preserving feature representation capacity; Mao et al. [44] developed a Residual Fast Fourier Transform with Convolution Block (ResFFT-Conv) module; and Mou et al. [45] transformed the Proximal Gradient Descent algorithm into a learnable deep architecture.

Beyond CNNs, other neural network architectures, including Recurrent Neural Networks (RNNs), Generative Adversarial Networks (GANs), and Feed-Forward Networks (FNNs), have demonstrated success in image deblurring. Zhang et al. [46] developed a Hybrid Deblur Net incorporating RNNs for non-uniform deblurring; Wang et al. [5] proposed a real-time deblurring algorithm utilizing GANs; and Kong et al. [47] developed a Frequency domain-based Self-Attention Solver (FSAS) to address the limitations of FFNs in image deblurring.

Despite their superior deblurring capabilities, neural networks face two significant limitations: (1) Their substantial data dependency means they require extensive training samples for optimal performance, resulting in generalization failures with distributionally shifted data. (2) Computational requirements are significant during both training and inference phases, particularly for architectures with numerous parameters.

## 3. Proposed Method

In this section, we present our improved sparse regularization and develop an effective deblurring algorithm based on this model.

### 3.1. Definition of Nonlinear Sparse Regularization

The nonlinear sparse regularization ($L_{N}$) is defined as:

(3) $$||\cdot |{|}_{N}=\frac{||\cdot |{|}_{p}}{||\cdot |{|}_{\infty }}$$

Given a corrupted signal A, we assume the latent sharp signal B is sparse. With a basic quadratic penalty, the objective energy function can be written as:

(4) $$B^{*}=\underset{B}{\arg\min}\|A-B{\|}_{2}^{2}+\lambda \|B{\|}_{N}$$

where λ denotes the regularization parameter, and k denotes the iteration level. Connecting to the definition of $L_{N}$ in (Equation (3)), Equation (4) can be rewritten as:

(5) $$B^{*}=\underset{B}{\arg\min}\|A-B{\|}_{2}^{2}+\lambda \frac{\|B{\|}_{p}}{\|B{\|}_{\infty }}$$

where $B^{*}$ denotes the result after this iteration level, while B denotes the result of the previous iteration level. The ${\left||\cdot |\right|}_{\infty }$ notation represents the infinity norm, which is mathematically defined as the maximum absolute value of a matrix, expressed as ${\left||\cdot |\right|}_{\infty }=max\{|\cdot |\}$. Building on previous successful practices [3,4,6,7], we utilize the infinity norm ($\|B{\|}_{\infty }$) of the signal from the previous iteration as a weighting factor to adjust the regularization parameter. To facilitate the solution of our model, we decompose the signals A and B into a series of independent subproblems:

(6) $${B_{i}}^{*}=\underset{{B_{i}}^{k}}{\arg\min}{\left|A_{i}-B_{i}\right|}_{2}^{2}+\lambda \frac{{\left|B_{i}\right|}^{p}}{\|B{\|}_{\infty }},$$

where i denotes the location of an element. The nonlinear sparse regularization term in Equation (6) presents a non-convex optimization problem, and following successful experiments, we are able to transform $L_{N}$ optimization into $L_{p}$ optimization with an adaptively adjustable regularization parameter. Based on the GST algorithm widely employed for $L_{p}$ norm optimization, we further develop an AGST algorithm. To illustrate the nonlinear sparse regularization, we abstract the $L_{N}$-related component in Equation (6) as a function:

(7) $$F(A_{i},B_{i})={\left(A_{i}-B_{i}\right)}^{2}+\lambda \frac{|B_{i}{|}^{p}}{\|B{\|}_{\infty }}.$$

**Figure 2:**
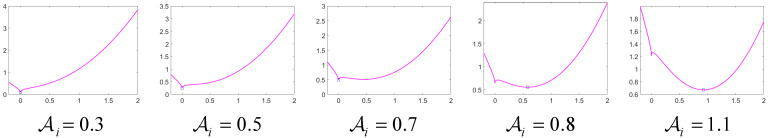
Curves of Equation (7) under different values of $A_{i}$, where *λ* = 2 and *p* = 0.5.

The curves of function F under different values of variable $A_{i}$ are displayed in the following figure:

The curves in Figure 2 illustrate the existence of a threshold τ: when $A_{i}$ falls below this threshold, the minimum value of the function in Equation (7) occurs at $B_{i}=0$; when $A_{i}$ exceeds this threshold, the function reaches its minimum at a non-zero value. These properties indicate that the threshold τ satisfies the following condition:

(8) $$F(\tau ,B_{i\tau })=F(0,B_{i\tau }),$$

(9) $$F’(\tau ,B_{i\tau })=0.$$

Here, $B_{i\tau }$ denotes the variable $B_{i}$ when the function in Equation (7) achieves its non-zero minimum, and F’ denotes the first-order derivative of F. These conditions yield the following solutions:

(10) $$B_{i\tau }={\left(\frac{\lambda (1-p)}{\|B{\|}_{\infty }}\right)}^{\frac{1}{2-p}},$$

(11) $$\tau ={\left(\frac{\lambda (1-p)}{\|B{\|}_{\infty }}\right)}^{\frac{1}{2-p}}+\frac{\lambda p}{2\|B{\|}_{\infty }}{\left(\frac{\lambda (1-p)}{\|B{\|}_{\infty }}\right)}^{\frac{p-1}{2-p}}.$$

As shown in Equation (11), since $\|B{\|}_{\infty }$ varies with the optimization target B, which directly relates to the threshold τ, the proposed AGST algorithm adapts its thresholding dynamically according to the intrinsic characteristics of input variables. The solution $B_{i}^{*}$ is expressed as:

(12) $${B_{i}}^{*}=AGST(A_{i},B,\lambda ,p).$$

The workflow of AGST is outlined in Algorithm 1.
**Algorithm 1:** The Adaptive Generalized Soft-Thresholding algorithm**input**: $A_{i}$, B, λ, *p*, *J*  $\tau ={\left(\frac{\lambda (1-p)}{\|B{\|}_{\infty }}\right)}^{\frac{1}{2-p}}+\frac{\lambda p}{2\|B{\|}_{\infty }}{\left(\frac{\lambda (1-p)}{\|B{\|}_{\infty }}\right)}^{\frac{p-1}{2-p}}$  **if**$|A_{i}|\leq \tau$    ${B_{i}}^{*}=0$  **else**    $b^{\left(0\right)}=|A_{i}|$    **for** *t* = 1, 2, …, *J*        $b^{\left(t\right)}=|A_{i}|-\frac{\lambda p}{\|B{\|}_{\infty }}{\left(b^{\left(t-1\right)}\right)}^{p-1}$        t←t+1    **end**    ${B_{i}}^{*}=\mathrm{sgn}(A_{i})b^{\left(J\right)}$  **end** **Output**: $B_{i}^{*}$


### 3.2. Deblurring Model and Optimization

This subsection describes the proposed deblurring model and its optimization procedure. In our formulation, we employ the $L_{2}$-norm, commonly utilized in traditional algorithms, to regularize the fidelity term and the blur kernel term. For image-related regularization terms, building upon previous successful approaches [9,10,11,13,14], we combine the $L_{N}$ gradient prior with the widely adopted $L_{0}$ gradient prior, thereby constructing a novel fast nonlinear sparse model. The complete model is expressed as:

(13) $$\underset{I,k}{\min}||\;I⊗k-B\;|{|}_{2}^{2}+\alpha ||\;\nabla I|{|}_{N}+\beta \|\;\nabla I{\|}_{0}+\gamma ||\;k\;|{|}_{2}^{2}$$

where ∇ denotes the gradient operators in vertical and horizontal dimensions (i.e., $=\{\nabla_{h},\nabla_{v}\}$); α, β, and γ denote the weight parameters. We solve Equation (13) by alternatively updating I and k with the other one held fixed. The sub-problems referring to I and k are given by:

(14) $$\underset{I}{\min}||\;I⊗k-B\;|{|}_{2}^{2}+\alpha ||\;\nabla I\;|{|}_{N}+\beta \|\;\nabla I{\|}_{0},$$

(15) $$\underset{k}{\min}||\;I⊗k-B\;|{|}_{2}^{2}+\gamma ||\;k\;|{|}_{2}^{2},$$

#### 3.2.1. Updating Latent Image I

The latent image I is updated while keeping the kernel k fixed. Since Equation (14) presents a highly non-convex problem, the HQS method is employed. Two auxiliary variables, u and g, are introduced to represent I in the second and third terms of Equation (14), respectively, transforming Equation (14) into:

(16) $$\underset{I,u,g}{\min}||\;I⊗k-B\;|{|}_{2}^{2}+\alpha \;||\;u\;|{|}_{N}+\beta \|\;g{\|}_{0}+\lambda_{1}||u-\nabla I|{|}_{2}^{2}+\lambda_{2}\|\;g-\nabla I{\|}_{2}^{2},$$

where $\lambda_{1}$ and $\lambda_{2}$ denote the penalty parameters. Similarly to Equation (13), Equation (16) is decomposed into three subproblems associated with I, u, and g:

(17) $$\underset{I}{\min}||\;I⊗k-B\;|{|}_{2}^{2}+\lambda_{1}||\;u-\nabla I\;|{|}_{2}^{2}+\lambda_{2}\|\;g-\nabla I{\|}_{2}^{2},$$

(18) $$\underset{u}{\min}\alpha ||\;u\;|{|}_{N}+\lambda_{1}||\;u-\nabla I\;|{|}_{2}^{2},$$

(19) $$\underset{g}{\min}\beta \|\;g{\|}_{0}+\lambda_{2}\|\;g-\nabla I\;{\|}_{2}^{2}.$$

**Solving** I**.** Equation (17) represents a classical quadratic optimization problem solvable via Fast Fourier Transform (FFT), with its closed-form solution expressed as:

(20) $$I=F^{-1}(\frac{\bar{F(k)}\cdot F(B)+\lambda_{1}\bar{F(\nabla )}\cdot F(u)+\lambda_{2}\bar{F(\nabla )}\cdot F(g)}{\bar{F(k)}\cdot F(k)+(\lambda_{1}+\lambda_{2})\bar{F(\nabla )}\cdot F(\nabla )}).$$

**Solving** u. Based on the definition in Equation (3), Equation (18) is reformulated as:

(21) $$\underset{u}{\min}\lambda_{1}\|\;u-\nabla I\;{\|}_{2}^{2}+\alpha \frac{\|\;u{\|}_{p}}{\|\;\nabla I_{pre}{\|}_{\infty }},$$

where $I_{pre}$ denotes the I obtained from the previous iteration level. Adopting the AGST algorithm, the solution for variable u is expressed as:

(22) $$u=AGST(\nabla I,\nabla I_{pre},\frac{\lambda_{1}}{\alpha },p).$$

**Solving** g. The objective function for the auxiliary variable g in Equation (19) represents an $L_{0}$ gradient prior [2]. The solution employs the unnatural distribution method, yielding the closed-form solution for *g*:

(23) $$g=\left\{\begin{array}{l} \nabla I\;,\;|g{|}^{2}>\frac{\beta }{\lambda_{2}}\\ 0\;,\;others \end{array}\right..$$

The principal steps for estimating the latent image I are summarized in Algorithm 2.
**Algorithm 2:** Latent image estimation**Input** Blurred image B, initialized k from the coarser level.   I←B, $\lambda_{1}\leftarrow \alpha$, $\lambda_{2}\leftarrow \beta$   **repeat**      Calculating u using Equation (22)      Calculating g using Equation (23)      Calculating I using Equation (20)      *λ*_1_←2*λ*_1_ *λ*_2_←2*λ*_2_   **until**
*λ*_1_ >*α_max_* **Output** Intermediate latent image I.

#### 3.2.2. Updating Blur Kernel *k*

The objective function for the blur kernel k presents a quadratic optimization problem similar to Equation (15). While Equation (15) emphasizes image intensity information, previous advanced methods [9,13,18,28] demonstrate that blur kernel estimation achieves higher accuracy when based on image gradient. Therefore, Equation (15) is modified into a gradient-based form:

(24) $$\underset{k}{min}\|\;\nabla I⊗k-\nabla B{\|}_{2}^{2}+\gamma \|\;k{\|}_{2}^{2},$$

and can be effectively solved through FFT:

(25) $$k=F^{-1}(\frac{\bar{F(\nabla I)}F(\nabla B)}{\bar{F(\nabla I)}F(\nabla I)+\gamma }).$$

The essential steps of blur kernel estimation are summarized in Algorithm 3.
**Algorithm 3:** Blur kernel estimation**Input** Blurred image B   Initialized k from the previous level of the image pyramid.      **while**
i≤max_iter **do**   Estimate I using Algorithm 2      Estimate k using Equation (25) **Output** Blur kernel k


## 4. Experimental Results

This section presents an evaluation of the proposed method using natural image datasets [8,48,49], real-world images, and a specific domain dataset [50], comparing it with several state-of-the-art methods, including traditional methods and deep learning methods. For all uniform image deblurring experiments, the parameters are set as α =0.0013, β=0.0023, γ =9, p=0.8, max_iter=5, and $\alpha_{max}=10^{5}$, and for fair comparison, the other algorithms utilize the default settings from the authors’ codes. Throughout the experiments, blur kernel estimation employed different blind deblurring methods, followed by the same non-blind deblurring method as the final step, and the model implementation used MATLAB R2022a with efficiency assessment conducted on an Intel Core i7-11800H CPU with 16GB RAM (Intel Corporation, Santa Clara, CA, USA).

### 4.1. Natural Images

This subsection demonstrates our method’s performance on two synthetic datasets from Levin et al. [8] and Sun et al. [49]. The restoration results undergo quantitative evaluation using three standard metrics: Peak Signal-to-Noise Ratio (PSNR), Structural Similarity Index Measure (SSIM), and cumulative error ratio. The mathematical definitions of each evaluation metric are expressed as follows:

(26) $$\begin{array}{c} PSNR=10\cdot \log_{10}(\frac{MAX_{S}^{2}}{MSE}),\\ s.t.MSE=\frac{1}{mn}\overset{m-1}{\underset{i=0}{\sum }}\overset{n-1}{\underset{j=0}{\sum }}[I(i,j)-I_{t}(i,j){]}^{2}, \end{array}$$

(27) $$SSIM(I,S)=\frac{\left(2\mu_{I}\mu_{S}+C_{1})(2\sigma_{IS}+C_{2}\right)}{\left(\mu_{I}^{2}+\mu_{S}^{2}+C_{1})(\sigma_{I}^{2}+\sigma_{S}^{2}+C_{2}\right)},$$

(28) $$error\;ratio=\frac{\|\;I_{t}-I{\|}_{2}^{2}}{\|\;I_{t}-I_{k}{\|}_{2}^{2}},$$

where I denotes the restored image, $I_{t}$ denotes the reference ground truth image used for quality assessment, and $I_{k}$ denotes the deblurred image using the real blur kernel. In Equation (26), m and n denote the size of an image, ${MAX}_{S}$ indicates the maximum pixel value of image S, and MSE means the mean squared error. In Equation (27), $\mu_{I}$ and $\mu_{S}$ denote the mean values of images I and S, $\sigma_{I}$ and $\sigma_{S}$ denote their standard deviation, $\sigma_{\mathrm{IS}}$ denotes the covariance between the two images, and $C_{1}$ and $C_{2}$ are two constants.

#### 4.1.1. Levin’s Dataset

**Figure 3:**
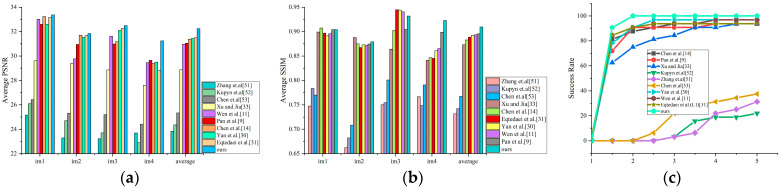
Quantitative evaluations of different algorithms on Levin et al.’s dataset. (**a**) Average PSNR; (**b**) average SSIM; (**c**) cumulative error ratio [9,11,14,30,31,33,51,52,53].

The initial evaluation utilizes the dataset reported by Levin et al. [8], comprising 32 blurred images generated from 4 images filtered with eight blur kernels, each maintaining a uniform resolution of 255 × 255 pixels. The PSNR, SSIM, and cumulative error ratio metrics of our method is compared with several state-of-the-art methods [9,11,14,30,31,33,51,52,53], with the results presented in Figure 3. As demonstrated in Figure 3a,b, the proposed model achieves superior average PSNR and SSIM metrics (32.234 dB in PSNR and 0.909 in SSIM), surpassing the $L_{e}$ model by 0.866 dB in PSNR and 0.024 in SSIM, respectively. Additionally, Figure 3c displays the cumulative error ratio curves of the comparative methods, with the results demonstrating that the proposed method consistently outperforms competing approaches, achieving a 90.625% success rate when the error ratio is ≤1.5, and a 100% success rate when the error ratio is ≤2.0.

**Figure 4:**
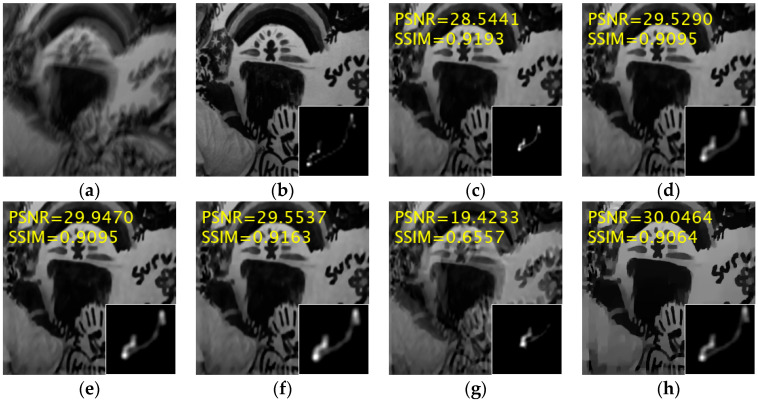
A visible comparison of the deblurring results for one image from the dataset [8]. (**a**) Blurred image; (**b**) Clear image; (**c**) Yan et al. [30]; (**d**) Pan et al. [9]; (**e**) Wen et al. [11]; (**f**) Eqtedaei et al. [31]; (**g**) Chen et al. [14]; (**h**) Ours.

Figure 4 presents a particularly challenging image—exhibiting a large blur kernel and complex texture details, posing significant difficulties for deblurring algorithms—alongside the restoration results of the compared methods, with corresponding PSNR and SSIM values annotated in the upper-left corner. The proposed method achieves superior kernel estimation accuracy and visual quality, producing the highest PSNR (30.046 dB).

#### 4.1.2. Sun’s Dataset

**Figure 5:**
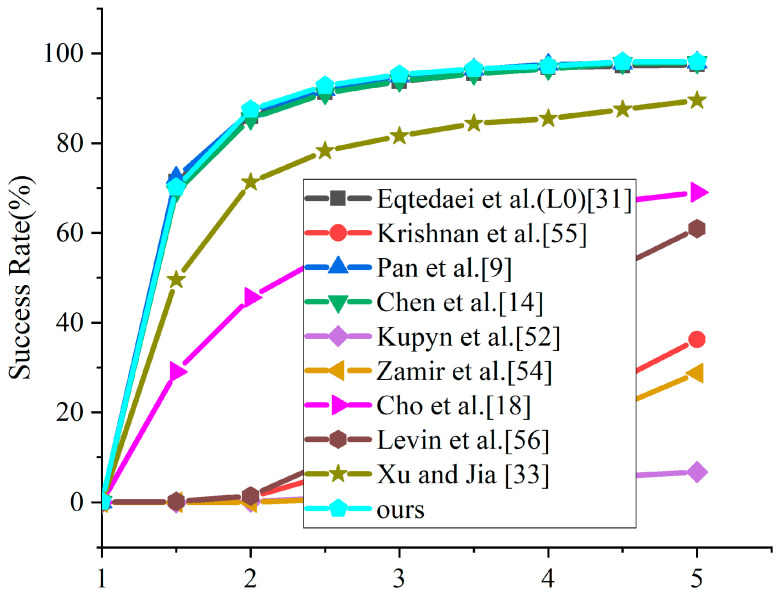
The cumulative error ratio statistics curve on Sun’s dataset [9,14,18,31,33,52,54,55,56].

To expand the comparative analysis, evaluation was conducted on Sun’s dataset [49], containing 640 high-resolution blurred images, using the cumulative error ratio as the comparison metric. Our method was evaluated against several established deblurring methods [9,14,18,31,33,52,54,55,56], with the quantitative results presented in Figure 5. For equitable comparison, an identical non-blind deblurring approach [57] was applied for all competing methods. As the figure illustrates, the proposed method achieves an 87.500% success rate when the error ratio is ≤2, exceeding Chen et al.’s [14] enhanced sparse model, which achieves an 85.469% success rate.

**Figure 6:**
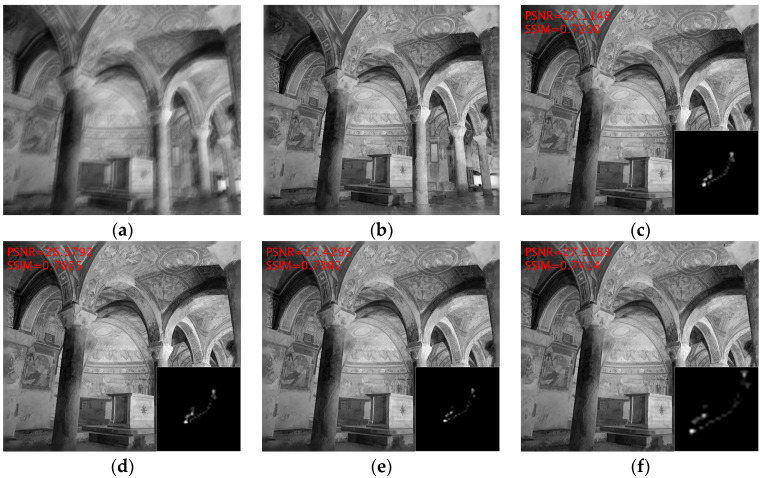
A visual example from Sun’s dataset [49]. (**a**) Blurred image; (**b**) Clear image; (**c**) Pan et al. [9]; (**d**) Eqtedaei et al. [31]; (**e**) Chen et al. [14]; (**f**) Ours.

Following an established protocol, a representative case is presented in Figure 6 to illustrate the advantages of the proposed method over comparative approaches, with quantitative metrics annotated in the upper-left corner. The selected example features an interior architectural space with intricate structural details, and the results demonstrate that the proposed method has superior blur kernel accuracy compared to alternative approaches. Notably, the method achieves a 0.489 dB PSNR improvement and 0.011 SSIM gain over the $L_{e}$ model proposed by Chen et al. [14].

### 4.2. Specific Images

With Section 4.1 having demonstrated the superior performance of the proposed method on natural images, this subsection presents the results of targeted experiments using representative scenarios from the dataset in [50], specifically evaluating performance on two distinct scenarios: human face images and text images.

#### 4.2.1. Human Face Images

**Figure 7:**
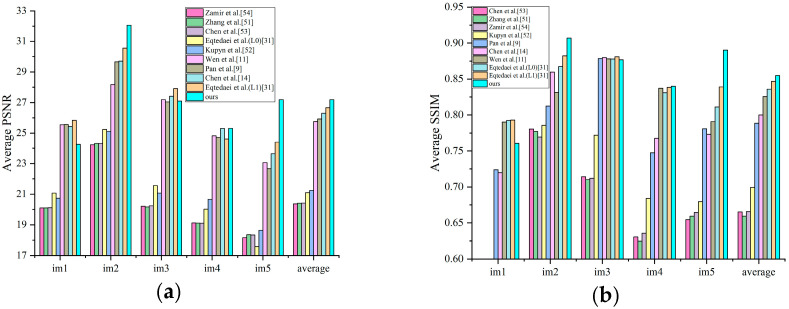
A quantitative comparison of restoration results for face images from the dataset of Lai et al. (**a**) Average PSNR; (**b**) average SSIM [9,11,14,31,51,52,53,54].

Face image processing constitutes a fundamental research area in this field, with images of faces presenting unique challenges due to their frequent absence of dominant structural information, complicating blur kernel estimation. Figure 7 presents quantitative comparison results of face images from the dataset in [50]. The results indicate the superior PSNR and SSIM metrics (27.638 dB PSNR and 0.860 SSIM) of the proposed method, showing an improvement of 1.337 dB in PSNR and 0.030 in SSIM compared to Chen et al.’s $L_{e}$ model [14].

**Figure 8:**
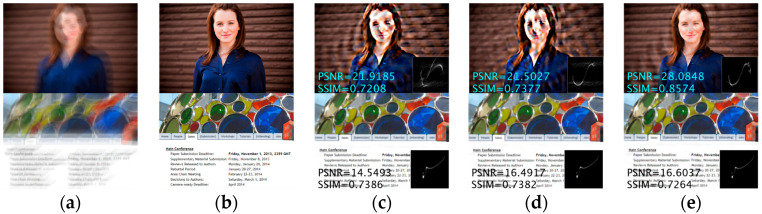
Visual examples of two classic scenarios (face and text) from the dataset of Lai et al. (**a**) Blurred image; (**b**) Clear image; (**c**) Eqtedaei et al. [31]; (**d**) Chen et al. [14]; (**e**) Ours.

The first row of Figure 8 presents a challenging face image example from the dataset [50], while column (b) displays the ground-truth sharp image. This example demonstrates that our method reconstructs the most accurate blur kernel while producing results with minimal ringing artifacts. Following standard practice, the PSNR and SSIM metrics are displayed in the under-left corner, indicating the substantial improvement achieved by the proposed method over the $L_{e}$ model, obtaining a PSNR of 28.085 dB.

#### 4.2.2. Text Images

**Figure 9:**
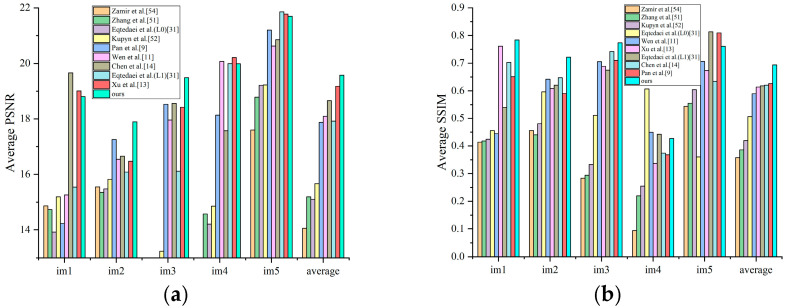
A quantitative comparison of restoration results for text images in dataset of Lai et al. (**a**) Average PSNR; (**b**) average SSIM [9,11,13,14,31,51,52,54].

Text image processing represents another significant application domain, different from other tasks in that these images predominantly contain two-tones that do not follow the heavy-tailed distribution of natural images, making text images particularly challenging for most deblurring methods. Figure 9 presents the average PSNR and SSIM for text images from the dataset in [50], indicating that our method achieves the highest quantitative evaluation metrics, surpassing the second-highest method by 0.533 dB in PSNR and 0.066 in SSIM. For visual comparison, the second row of Figure 8 illustrates an exemplar text image from the dataset [19], containing abundant image details. The comparative experimental results demonstrate that our method generates reconstructed results with superior detail preservation. The quantitative analysis metrics indicated in the upper-left corner reveal a PSNR improvement of 0.374 dB compared to other methods.

### 4.3. Comparison Against Deep Learning Methods

**Figure 10:**
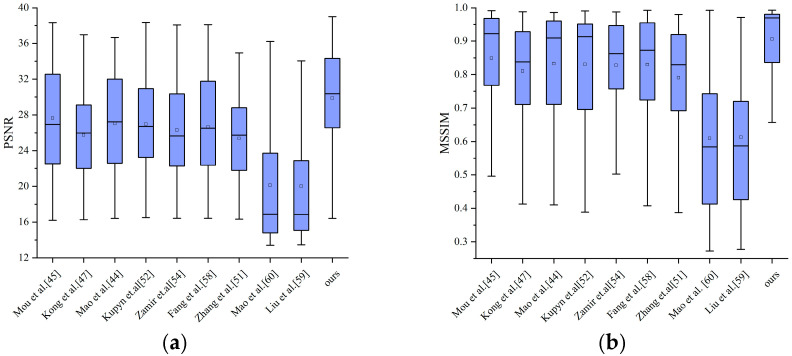
A quantitative comparison of our method with other deep-learning methods on the dataset proposed by Köhler et al. (**a**). PSNR; (**b**). MSSIM.

The recent decade has witnessed rapid advancement in deep learning, leading to numerous deep learning-based deblurring methods being proposed. To validate the effectiveness of our approach, we performed comparative experiments with several state-of-the-art deep learning models [44,45,47,51,52,54,58,59,60] on dataset proposed by Köhler et al. [48]. The quantitative analysis results, presented in Figure 10, demonstrate that our model achieves superior performance compared to several deep learning approaches in terms of both PSNR and Mean SSIM (MSSIM), surpassing the best-performing deep learning model [45] by 2.237 dB and 0.057, respectively.

**Figure 11:**
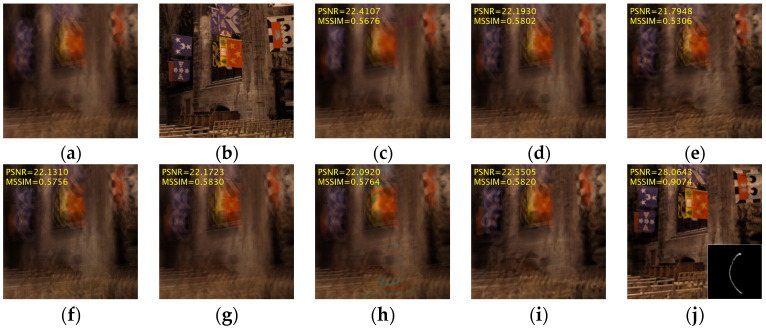
A visual example from the dataset proposed by Köhler et al. (**a**) Blurred image; (**b**) clear image; (**c**) Kupyn et al. [52]; (**d**) Zamir et al. [54]; (**e**) Zhang et al. [51]; (**f**) Fang et al. [58]; (**g**) Mao et al. [44]; (**h**) Kong et al. [47]; (**i**) Mou et al. [45]; (**j**) Ours.

For qualitative evaluation, we illustrate a challenging example from the dataset in Figure 11. The results indicate that most deep learning methods encounter difficulties in producing satisfactory restoration results when processing images with large blur kernels. Our method, however, maintains accurate reconstruction quality even in this challenging case, achieving a PSNR of 28.064 dB and an MSSIM of 0.907.

### 4.4. Real-World Images

**Figure 12:**
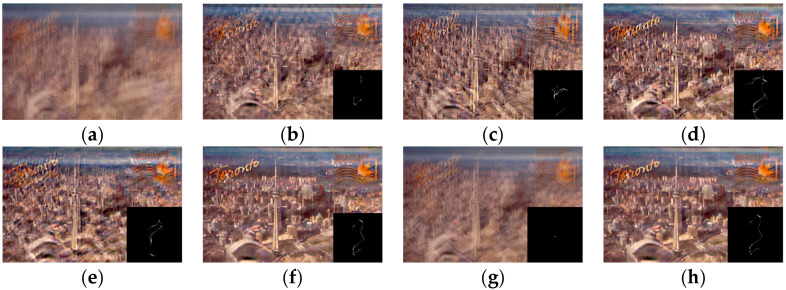
A comparative analysis of restoration results for a real-world blurred image. (**a**) Blurred image; (**b**) Pan et al. [9]; (**c**) Chen et al. [10]; (**d**) Xu et al. [13]; (**e**) Eqtedaei et al. [31]; (**f**) Wen et al. [11]; (**g**) Chen et al. [14]; (**h**) Ours.

Following evaluation on synthetic datasets, we tested our algorithm using real-world blurred images, inputs that present greater randomness and uncertainty compared to synthetic images, thus imposing higher requirements on algorithmic stability and adaptability. Figure 12 illustrates an example of a real-world blurred image, featuring rich structural details and exhibiting relatively complex blur kernel size and motion trajectory characteristics. To ensure fair comparison, we applied the same non-blind image deblurring algorithm [8] with identical parameter settings throughout all experiments, and the other methods’ restoration parameters for other methods were configured using the combinations published by their respective authors. The results demonstrate that our proposed fast nonlinear sparse model achieves the most accurate blur kernel estimation among all compared methods, producing a final restored image with superior detail preservation and minimal ringing artifacts.

## 5. Analysis and Discussion

In this section, we analyze a series of ablation experiments to systematically validate the effectiveness of our proposed fast nonlinear sparse model, accompanied by comprehensive discussions on key parameter influences and computational efficiency.

### 5.1. The Effectiveness of the Fast Nonlinear Sparse Model

This section presents a series of ablation experiments conducted to validate the effectiveness of the proposed fast nonlinear sparse model, evaluating various combinations of regularization norms commonly employed in deblurring methods, with quantitative results illustrated in Table 1. Specifically, columns 1–3 present the quantitative deblurring evaluations for three distinct regularization method–$L_{1}/L_{2}$, $L_{1}/L_{\infty }$, and $L_{p}/L_{\infty }$; column 4 presents the average PSNR and SSIM obtained by standalone $L_{0}$-regularization; and columns 5–7 demonstrate the corresponding performance when these norm-ratio-based regularizations are coupled with the $L_{0}$ norm.

The comparative analysis reveals three significant findings: First, within columns 1–3, the proposed nonlinear sparse regularization ($L_{p}/L_{\infty }$) exhibits substantial advantages over other norm-ratio-based regularizations. Compared to the $L_{1}/L_{\infty }$ prior, the proposed $L_{p}/L_{\infty }$ prior achieves an average PSNR improvement of 0.591 dB, indicating superior performance over other nonlinear coupled norm priors. Second, the $L_{0}$ coupled versions (columns 5–7) achieve markedly better performance than their standalone counterparts (columns 1–3). Incorporating the $L_{0}$ prior yields an average improvement of 1.629 dB in PSNR and 0.038 in SSIM, which demonstrates that the $L_{0}$ regularization prior significantly enhances algorithm performance. Third, the results in the last four columns indicate that combining nonlinear coupled norm priors with the $L_{0}$ prior enhances the deblurring algorithm’s performance more effectively than using the $L_{0}$ prior alone, achieving PSNR improvements of 0.184 dB, 0.091 dB, and 0.679 dB, respectively. Furthermore, the proposed fast nonlinear sparse model, integrating the $L_{N}$ prior with the $L_{0}$ prior, achieves optimal deblurring performance with a 32.234 dB PSNR and 0.909 SSIM.

### 5.2. Effect of Main Parameters

**Figure 13:**
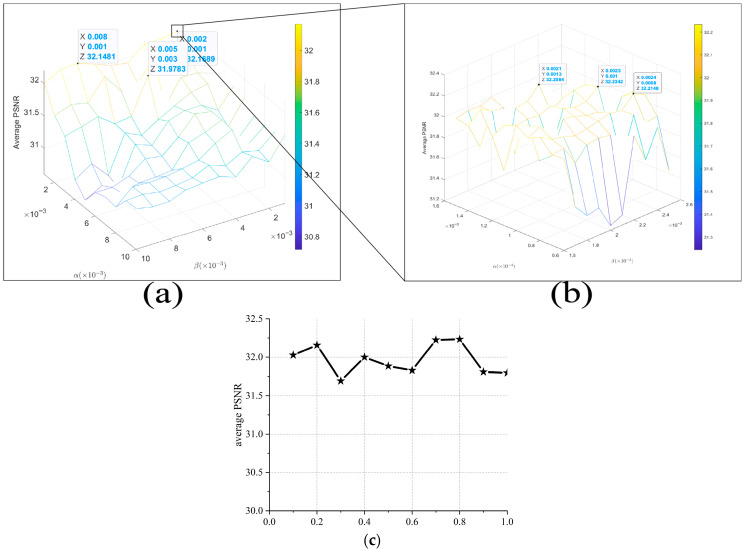
Parameter search grids. (**a**,**b**) Primary and secondary search grids for *α* and *β*; (**c**) independent search grid for parameter *p*.

The proposed fast nonlinear sparse model incorporates four key parameters: α, β, γ, and p. The parameter optimization process consists of two stages: first, a two-dimensional grid search for the key parameters, α and β, utilizing the average PSNR as the selection criterion, and second, a separate search grid for parameter p, given its relative independence, as illustrated in Figure 13c.

First, based on existing empirical knowledge, we construct a grid search for parameters α and β over the interval [$1\times 10^{-3}$, $10\times 10^{-3}$] with a step size of $1\times 10^{-3}$, as illustrated in Figure 13a, where the boxed coordinate indicates the position yielding the maximum average PSNR. This primary grid serves to identify the approximate optimal ranges for α and β. Subsequently, we perform a secondary grid search within the identified optimal range from the initial screen, employing a refined step size of $10^{-4}$ (Figure 13b), enabling the precise determination of the final parameter values.

**Figure 14:**
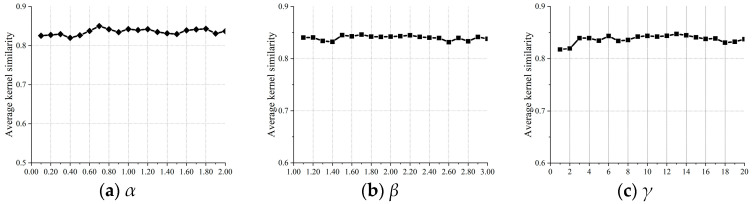
Impact of three key parameters on our model’s restoration performance.

Parameter stability is essential for robust optimization-based deblurring, and that of the fast nonlinear sparse model was evaluated using blur kernel similarity (Figure 14). Parameters α and β demonstrate stable performance across the refined grid range illustrated in Figure 13b, with kernel similarity variances of $5.62\times 10^{-5}$ and $1.78\times 10^{-7}$, respectively. All three parameters show minimal fluctuations in their similarity curves, confirming stable performance within reasonable ranges. Parameter γ exhibits particularly low sensitivity, maintaining stable kernel similarity across its 3–20 operating range (Figure 14c).

### 5.3. Runtime Analysis

Computational efficiency serves as a crucial metric for evaluating deblurring algorithm performance, whit shorter durations indicating higher efficiency. In this experiment, we assess the computational time of our algorithm across three distinct image resolutions (255 × 255, 600 × 600, 800 × 800 pixels), with testing conditions controlled by maintaining a fixed blur kernel size of 27 × 27 throughout the trials. The comprehensive timing results are presented in Table 2.

We compared runtime results between our method and several classical patch-based deblurring approaches [9,10,11,13,31]. As demonstrated in Table 2, non-overlapping patch priors [11,13] show substantial efficiency improvements over overlapping patch design priors [9,10,31], with our method achieving an approximately 50% runtime reduction compared to standard non-overlapping patch-based methods. This enhancement results from its pixel-wise optimization strategy, which eliminates patch extraction requirements in each iteration.

### 5.4. Limitations

**Figure 15:**
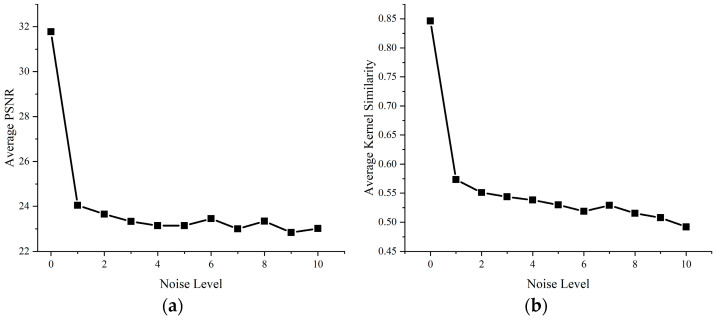
Quantitative analysis of our method under different noise levels. (**a**) the average PSNR; (**b**) the average Kernel Similarity.

**Figure 16:**
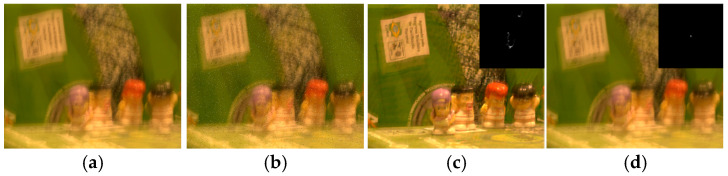
An example of noise image deblurring. (**a**) Noise-free blurred image; (**b**) Blurred image with salt-and-pepper noise; (**c**) Noise-free deblurring result; (**d**) Deblurring result with salt-and-pepper noise.

**Figure 17:**
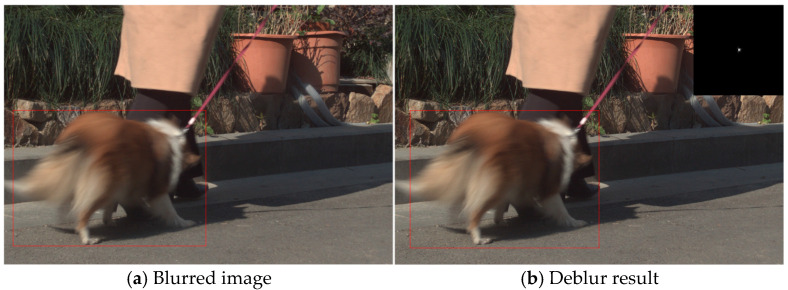

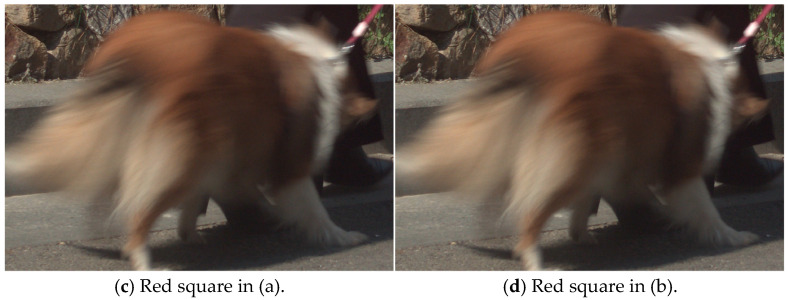
An example of a highly non-uniform locally blurred image.

In the previous sections, we evaluated the superior performance and computational efficiency of our proposed nonlinear sparse model compared to existing state-of-the-art methods using both synthetic datasets and real-world images. However, our model still exhibits certain limitations. First, due to its pixel-wise computation approach during optimization, it demonstrates poor resistance to salt-and-pepper noise; as shown in Figure 15 and Figure 16, restoration performance deteriorates significantly when such noise is present. Additionally, our model performs weakly when handling locally blurred images, such as that in Figure 17.

## 6. Conclusions

This paper introduces a novel nonlinear sparse regularization ($L_{N}$-regularization) based on the nonlinear coupling of the $L_{p}$ and $L_{\infty }$ norms, and to facilitate effective optimization, an AGST algorithm is developed. Through the integration of the $L_{N}$ and $L_{0}$ regularization priors, this research establishes a fundamentally new fast nonlinear sparse model. Statistical analyses demonstrate that $L_{N}$ regularization achieves optimal sparsity. Comprehensive experiments on synthetic datasets and real-world blurred images validate that our fast nonlinear sparse model delivers superior deblurring performance. Quantitative results show that the proposed model achieves approximately 1 dB higher PSNR and 0.04 better SSIM values compared to state-of-the-art optimization-based deblurring methods, further reducing the computational time by 50% compared to conventional patch-based approaches due to its pixel-wise optimization strategy.

## Figures and Tables

**Table 1:** Quantitative analysis of the ablation experiment.

	$\frac{\|\cdot {\|}_{1}}{\|\cdot {\|}_{2}}$	$\frac{\|\cdot {\|}_{1}}{\|\cdot {\|}_{\infty }}$	$\frac{\|\cdot {\|}_{p}}{\|\cdot {\|}_{\infty }}$	$\|\cdot {\|}_{0}$	$\frac{\|\cdot {\|}_{1}}{\|\cdot {\|}_{2}}+\|\cdot {\|}_{0}$	$\frac{\|\cdot {\|}_{1}}{\|\cdot {\|}_{\infty }}+\|\cdot {\|}_{0}$	$\frac{\|\cdot {\|}_{p}}{\|\cdot {\|}_{\infty }}+\|\cdot {\|}_{0}$
Average PSNR	28.481	30.829	31.420	31.555	31.739	31.646	32.234
Average SSIM	0.811	0.884	0.894	0.889	0.895	0.895	0.909

**Table 2:** Comparison of runtime performance (in seconds) across three different resolutions for various methods.

	255 × 255	600 × 600	800 × 800
Pan et al. [9]	63.652	327.051	659.056
Wen et al. [11]	10.229	22.034	59.721
Xu et al. [13]	9.321	35.021	82.246
Chen et al. [10]	33.041	179.554	327.993
Eqtedaei et al. [31]	27.079	114.625	198.729
Ours	4.657	20.871	35.443

## Data Availability

The data presented in this study are available on request from the corresponding author.
